# Measuring of strain parameters reflects changes of right ventricular function before and after thrombolytic therapy in patients with acute pulmonary embolism

**DOI:** 10.1007/s10554-022-02626-8

**Published:** 2022-05-20

**Authors:** Shen-Yi Li, Yi Zhang, Ting-Ting Shen, Tian-Tian Lu, Xi Li

**Affiliations:** Department of Ultrasonography, The People’s Hospital of Hunan Province, No. 61 Jiefang West Road, Changsha, 410005 China

**Keywords:** Speckle tracking echocardiography (STE), Pulmonary embolism, Pulmonary hypertension, Right ventricular, Strain

## Abstract

Strain parameters on speckle tracking echocardiography (STE) have been proposed as effective indexes for evaluating right ventricular (RV) function. This pilot study investigated the role of STE-derived strain parameters in assessing global and regional RV myocardial mechanical changes in patients with acute pulmonary embolism (PE) before and after thrombolytic therapy. In this case–control study, a total of 73 PE patients, 34 with pulmonary hypertension (PH) and 39 without PH, who underwent thrombolytic therapy were included. Healthy volunteers were included as controls. The peak longitudinal systolic strain (PLSS) and time to PLSS (TTP) for the global and regional RV were analyzed by STE software immediately before and 14 days after thrombolytic therapy. Changes in STE-derived strain parameters and conventional ultrasound parameters were compared. PLSS and TTP decreased before treatment in PE patients compared with measurements in the control group, particularly in those with PH. Also, the strain parameters decreased more significantly for the free wall than for the septum wall (P < 0.05). Moreover, the RV diastolic diameter (RVDD) and RV/left ventricular (LV) diameter ratio increased, while RV fraction shortening (RVFS), RV fractional area change (RVFAC), tricuspid regurgitation pressure gradient (TRPG), and tricuspid annular peak systolic excursion (TAPSE) decreased (P < 0.05). The global strain parameters for the RV were positively correlated with RVDD and RV/LV diameter ratio, but negatively correlated with RVFS, RVFAC, TRPG, and TAPSE (P < 0.05). After treatment, the strain parameters differed significantly between PE patients with PH and controls but did not differ between PE patients without PH and controls. STE-derived parameters are effective for detecting changes in global and regional RV function in PE patients with or without acute PH.

## Introduction

Despite significant improvements in diagnostic and therapeutic strategies in recent years, pulmonary embolism (PE) remains a severe pulmonary vascular disease with a high incidence and mortality. For patient with PE, subsequent right ventricular (RV) dysfunction has become an important cause of death, and early appearance of RV dysfunction after PE has been confirmed as an independent risk factor of mortality in these patients [1]. Accordingly, methods for early detection of RV dysfunction in PE patients are of important clinical significance. However, due to the complex anatomical structure and the influence of the respiratory cycle on its diastolic blood flow filling, evaluation of RV function is relatively difficult. Pulmonary angiography is currently regarded as the gold standard for the diagnosis of PE. However, this examination is invasive and cannot assess RV function [2]. Echocardiography has become the preferred imaging method for RV function evaluation because of its good repeatability, noninvasive nature, and high accuracy, but it has a low sensibility and specificity in the diagnosis of Pulmonary Embolism. Speckle tracking echocardiography (STE) is a new ultrasonic quantitative evaluation method based on cardiac mechanics and myocardial deformation that can be applied to effectively evaluate both global and regional myocardial function [3]. However, the efficacy of STE-derived strain parameters for the evaluation of RV function has yet to be proven, particularly in patients with PE who have undergone thrombolytic therapy. In the present study, we aimed to evaluate the dynamic changes of the long axis strain parameters of the RV in PE patients before and after thrombolytic therapy. Additionally, we analyzed the correlations of these parameters with conventional ultrasonic RV parameters and the potential effects of the comorbidity of pulmonary hypertension (PH) on these parameters.

## Methods

### Study population and design

In this case–control study, a total of 73 patients (41 males and 32 females) diagnosed with PE in People’s Hospital of Hunan Province between March 1, 2016 and March 1, 2020 were included in this study (41 males and 32 females). Another 40 healthy volunteers without PE were included as controls (22 males and 18 females). Diagnosis of PE was performed in accordance with the 2014 European Heart Society guidelines for PE Management [4]. According to the cut-off point value for pulmonary artery pressure of 35 mmHg [5], PE patients were divided into two groups: PE without PH (pulmonary artery pressure < 35 mmHg) and PE with PH (pulmonary artery pressure ≥ 35 mmHg). Patients with acute coronary syndrome, obvious valvular disease, congenital heart disease, lung or kidney disease, or malignant arrhythmia were excluded from this study. Patients with a history of cardiac surgery or cases for which the quality of ultrasound images was poor were also excluded from this study. We used Wells score to assess the risk of pulmonary embolism (Table 1). Unfortunately, we did not count the number of patients excluded according to the exclusion criteria, but we counted a total of 3 patients who were excluded from the study group due to poor image quality of ultrasound imaging. The demographic and basal clinical characteristics of PE patients and controls were retrieved from the hospital database. This study was approved by the Ethics Committee of People’s Hospital of Hunan Province. All procedures performed in studies involving human participants were in accordance with the ethical standards of the institutional and national research committee and with the 1964 Declaration of Helsinki and its later amendments or comparable ethical standards. All participants provided written informed consent. Patients with a confirmed diagnosis of PE without contradictions underwent thrombolytic therapy via intravenous administration of Urokinase 20,000 IU/kg within 2 h. All patients were treated with thrombolytic therapy within 24 h from the onset of symptoms, and the first echocardiography was performed within 2 h before thrombolysis. The second echocardiography was performed on the 14th day after thrombolytic therapy.

**Table 1:** Clinical characteristics and conventional ultrasound parameters of healthy control and PE patients with and without PH before treatment

	Healthy control (n = 40)	PE without PH (n = 39)	PE with PH (n = 34)
Age, years (mean ± SD)	55.95 ± 8.84	57.36 ± 7.22	59.42 ± 9.28
Height (cm)	162.43 ± 9.12	161.07 ± 8.57	162.42 ± 7.13
Weight (kg)	59.33 ± 7.37	62.15 ± 8.47	61.05 ± 9.31
HR (bpm)	71.10 ± 10.25	72.55 ± 8.46	74.19 ± 12.94
Drug history [n(%)]	11(27.5)	15(38.46)^△^	19(55.88)^△^
Systolic pressure	114 ± 23.27	132.35 ± 20.32^△^	141.51 ± 25.66^△#^
Diastolic pressure	85 ± 10.76	95.46 ± 17.34^△^	98.33 ± 19.67^△#^
Hypertension [n(%)]	8(20)	11(28.20)*	14(41.17)^△^
Diabetes [n(%)]	9(22.5)	10(25.64)*	13(38.23)^△^
Dyspnea [n(%)]	–	33(84)	33(97)
Chest tightness [n(%)]	–	28(72)	30(88)
Hemoptysis [n(%)]	–	25(64)	28(83)
Syncope [n(%)]	–	21(54)	25(74)
Wells score	–	4.1 ± 2.84	4.72 ± 1.63
RVDD (mm)	29.19 ± 6.76	32.59 ± 7.82*	35.77 ± 8.92^△#^
RV FS (%)	33.52 ± 5.19	29.80 ± 6.79*	23.11 ± 6.37^△#^
RV FAC (%)	37.31 ± 7.42	35.91 ± 6.43*	30.45 ± 7.37^△#^
RADD (mm)	32.27 ± 4.11	34.14 ± 6.13*	37.24 ± 6.85^△^
RV/LV (%)	0.75 ± 0.44	0.88 ± 0.74*	1.18 ± 0.43^△#^
TAPSE (cm)	2.49 ± 0.48	1.83 ± 0.33^△^	1.62 ± 0.64^△#^

Data are expressed as $\overline{x} \pm S$

*RVDD* right ventricular diastolic diameter, *RVFS* right ventricular fraction shortening, *RVFAC* right ventricular fractional area change, *RADD* right atrium diastolic diameter, *RV/LV* right ventricular to left ventricular diameter ratio, *TAPSE* tricuspid annular peak systolic excursion

Compared to control group, *P < 0.05, ^△^*P* < 0.01

Compared to PE without PH group, ^#^*P* < 0.05 inPE with PH group

### Echocardiography

Echocardiography was performed using a GE Vivid E9 ultrasound system with a M5S transducer (GE Medical Healthcare, China) at a frame rate of ≥ 50 frame/sec. During the examination, participants were in a calm state and kept in the left lateral position or supine position. If the condition of the patient was not satisfactory for the examination, we waited until the general condition of the patient was relatively suitable for the examination. Conventional parameters for RV function evaluation, including RV diastolic diameter (RVDD), RV fraction shortening (RVFS), RV fractional area change (RVFAC), right atrium diastolic diameter (RADD), and RV/left ventricular (LV) diameter ratio were measured and calculated in the standard apical four chamber section. Tricuspid annular peak systolic excursion (TAPSE) was measured by M-mode ultrasound. Tricuspid regurgitation pressure gradient (TRPG) was measured by continuous-wave (CW) Doppler, and the pulmonary arterial systolic pressure (PASP) was subsequent calculated according to the Bernoulli equation: PASP = 4 × (velocity of tricuspid regurgitation) ^2^ + average RA pressure.

### Speckle tracking echocardiography (STE)

**Fig. 1:**
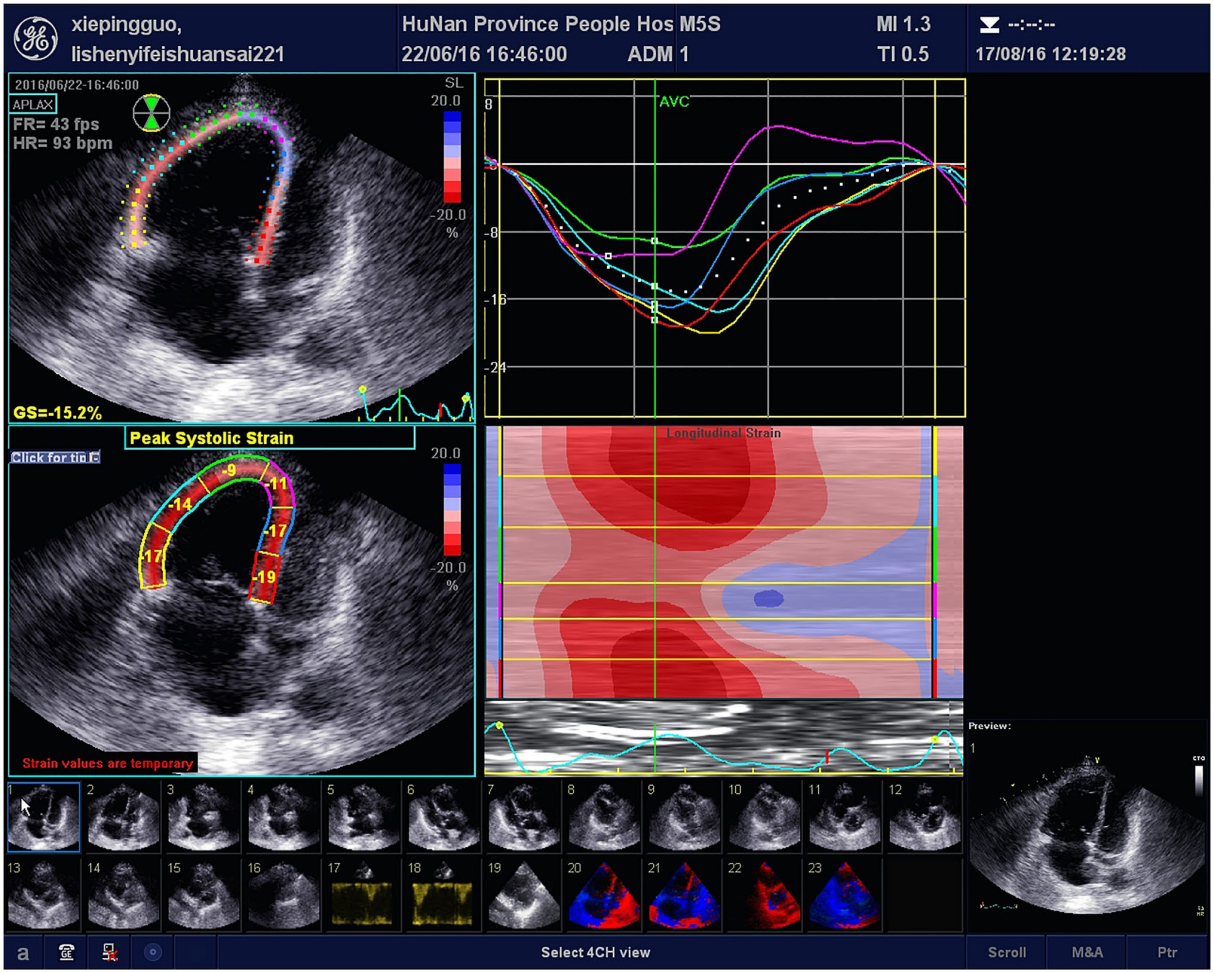
Representative images of RV PLSS in a patient with PE before the thrombolytic therapy

**Fig. 2:**
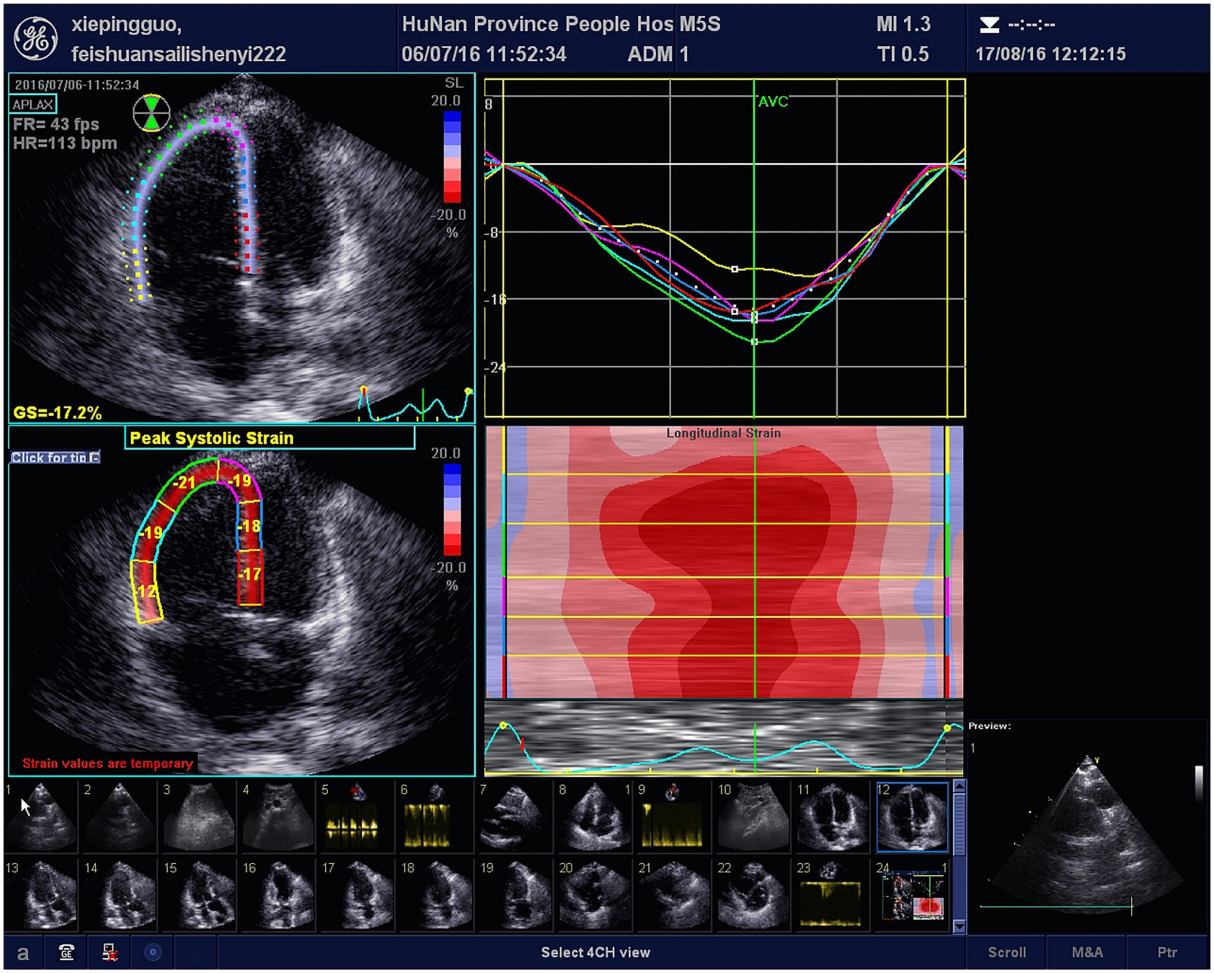
Representative images of RV PLSS in a patient with PE after treatment with thrombolytic therapy

All participants were instructed to hold their breath at the end of exhalation (when the patient’s condition allowed, we made our best effort to collect images suitable for STE analysis), and the apical RV focused view dynamic images were recorded for three consecutive cardiac cycles with a stable heart rhythm. Then the strain parameters were analyzed by STE software configured by the instrument. With the inner wall boundary of the RV traced by spots at the end of systolic period, the software automatically calculates the displacement of the spots in the region of interest in each frame of the whole cardiac cycle, and then the longitudinal strain curves for the six segments of the RV (the basal segment, the middle segment and the apex segment of the free wall and the intermediate wall) are obtained. The degree of myocardial deformation and peak longitudinal strain represent the percentage of longitudinal shortening of each segment of interest in systole compared with that in diastole. The time needed to reach the peak strain is time to peak strain. The time of QRS wave initiation is taken as the reference point. Strain indexes including peak longitudinal strain (PLSS) and time to PLSS (TTP) of the global RV as well as the six segments of the RV (basal free wall, mid free wall, apical free wall, basal septum, mid septum, and apical septum) were obtained. The global PLSS and TTP of the RV were calculated as the average values of the above six segments. Typical images for STE measurements before and after thrombolytic therapy are shown in Figs. 1 and 2.

### Reproducibility analysis

To determine the repeatability of RV strain parameters evaluated by STE, the analyses were repeated in a randomly selected group of 30 study subjects by two examiners with the same qualifications, and 30 cases who were randomly selected from the subjects were analyzed by the same examiner at an interval of two weeks.

Intraclass correlation coefficients (ICC) were used to assess inter-observer reliability and test–retest reliability. The investigators were blinded to the results of all previous measurements. The calculated ICCs indicated excellent inter-observer reliability (0.72–0.84%) and test–retest reliability (0.78–0.89%).

### Statistical analysis

Continuous data were presented as mean ± standard deviation (SD). The Shapiro–Wilk test was used to determine the distribution of data in the PE and control groups. The t test was used to identify differences in clinical characteristics and conventional ultrasound parameters between healthy controls and PE patients with PH and between the healthy controls and PE patients without PH before treatment. Also, the t test was used to identify differences in PLSS and TTP for the global RV and six segments of the RV between healthy controls and PE patients with PH, between healthy controls and PE patients without PH before and after treatment. Qualitative data are expressed as percentages (%) and were compared using the χ^2^ test. A P value < 0.05 was regarded as statistically significant. Pearson’s correlation analysis was performed to evaluate the correlations between the strain parameter PLSS and conventional ultrasonic parameters. All statistical analyses were performed using SPSS 13.0 software (SPSS Inc, Chicago, IL, USA).

## Results

### Clinical characteristics of the included patients

A total of 73 PE patients (44 males and 29 females) with an average age of 58.37 ± 8.12 years (range: 44–72 years) and 40 controls (22 males and 18 females) with an average age of 55.95 ± 8.84 years (range: 40–70 years) were enrolled in this study. Among the 73 patients, 39 were without PH at the time of PE diagnosis, while the remaining 34 had PH. No significant differences in the demographic and baseline clinical characteristics were observed between the groups (Table 1).

### Conventional ultrasonic parameters

Compared with the healthy controls, patients with PE before treatment had significantly higher RVDD and RV/LV diameter ratio and significantly lower RVFS and RVFAC (P < 0.05; Table 1), whereas RADD did not significantly differ between the groups (P > 0.05; Table 1). Moreover, PE patients had a significantly lower TAPSE before treatment as compared to controls (P < 0.01; Table 1). After treatment, the RVDD and RV/LV diameter ratio of PE patients were significantly decreased (P < 0.05; Table 1), whereas the RVFS, RVFAC, and TAPSE were significantly increased (P < 0.05). After treatment, the above parameters were no longer significantly different between PE patients without PH and controls, but remained significantly different between PE patients with PH and controls (P < 0.05; Table 1).

### Strain parameters

**Fig. 3:**
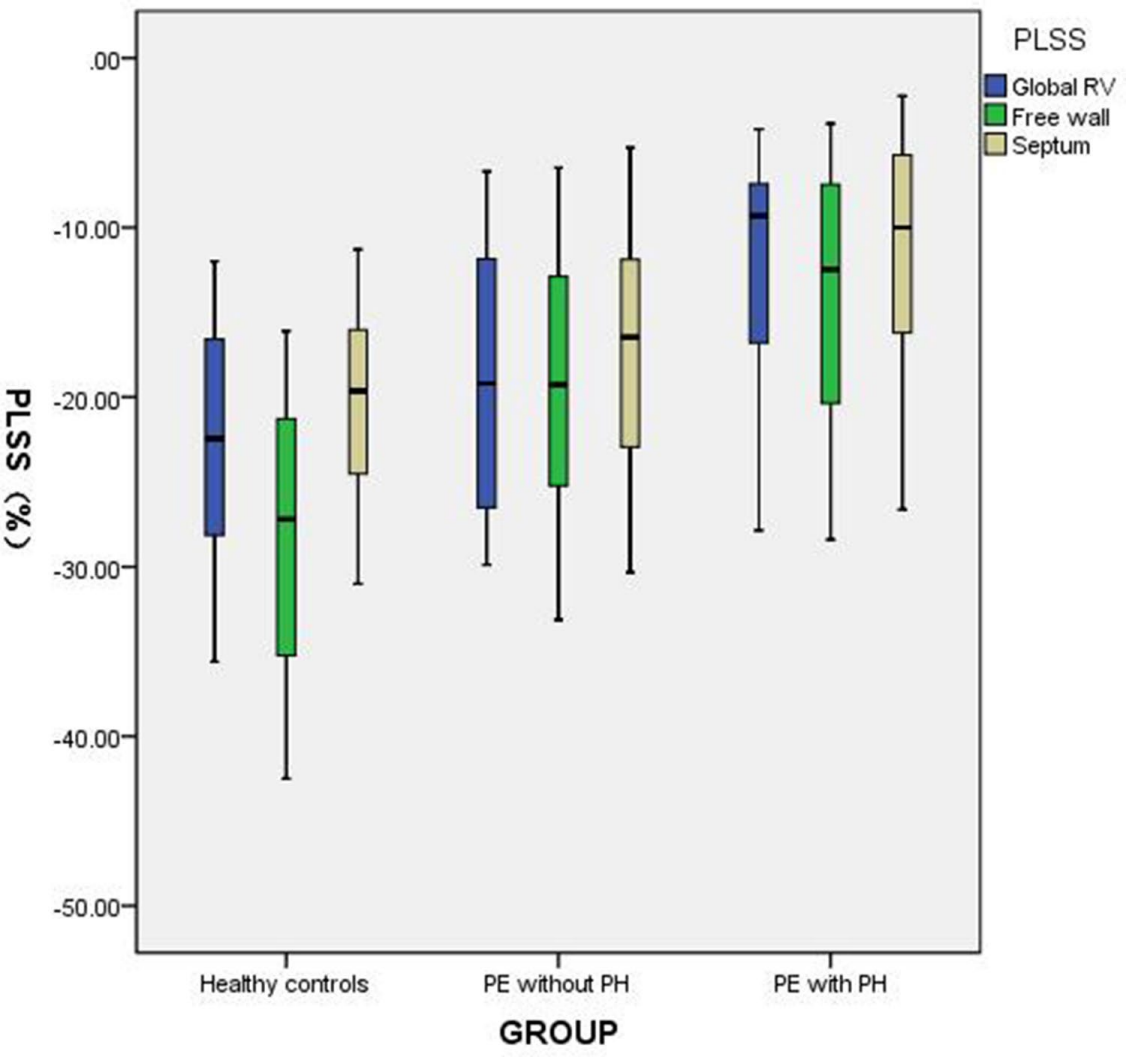
PLSS of the global RV, free wall and septum in healthy controls, PE patients without PH and PE patients with PH

Before treatment, the absolute values for PLSS and TTP of the global RV and each segment of RV in the PE groups were lower than those in the control group (P < 0.05). The degree of strain parameter reduction in PE patients with PH was greater than that in PE patients without PH. In each segment of the RV, the strain parameters for the free wall segment decreased more significantly than those for the septum wall in the PE groups. After treatment, no significant differences in strain parameters were observed between the without PH group and the control group (P > 0.05; Tables 2 and 3). The strain parameters in PE patients with PH after treatment were still lower than those in the control group (P < 0.05). However, the recovery amplitudes for the global and regional stain parameters of the RV in PE patients with PH were larger than in those without PH (Fig. 3).

**Table 2:** PLSS for the global RV and six segments of the RV in healthy control and PE patients with and without PH before and after treatment

PLSS (%)	Healthy control (n = 40)	PE without PH before treatment (n = 39)	PE with PH before treatment (n = 34)	PE without PH after treatment (n = 39)	PE with PH after treatment (n = 34)
Global	− 22.89 ± 7.17	− 18.67 ± 7.37^△^	− 12.37 ± 6.68^△^	− 21.65 ± 7.98	− 18.38 ± 7.83*
Basal free wall	− 29.89 ± 8.07	− 21.15 ± 8.39^△^	− 15.00 ± 8.69^△^	− 27.63 ± 10.39	− 25.41 ± 9.63*
Mid free wall	− 29.01 ± 7.43	− 19.09 ± 7.49^△^	− 13.96 ± 6.98^△^	− 26.16 ± 7.31	− 21.89 ± 7.81*
Apical free wall	− 25.51 ± 9.07	− 17.18 ± 7.07^△^	− 13.27 ± 6.63^△^	− 23.30 ± 8.55	− 21.15 ± 7.22*
Basal septum	− 21.02 ± 5.98	− 16.75 ± 8.89^△^	− 12.51 ± 6.98^△^	− 19.56 ± 7.58	− 17.72 ± 6.38*
Mid septum	− 20.35 ± 5.25	− 16.83 ± 7.04^△^	− 10.68 ± 5.67^△^	− 18.17 ± 6.85	− 16.28 ± 5.96*
Apical septum	− 19.59 ± 5.72	− 16.42 ± 4.51^△^	− 11.84 ± 7.98^△^	− 19.09 ± 8.21	− 16.79 ± 6.26*

Data are expressed as $\overline{x} \pm S$

*RV* right ventricular, *PE* pulmonary embolism, *PH* pulmonary hypertension, *PLSS* peak longitudinal systolic strain

Compared to control group, *P < 0.05, ^△^*P* < 0.01

**Table 3:** TTP for the global RV and six segments of the RV in healthy controls and PE patients with and without PH before and after treatment

TTP (ms)	Healthy control (n = 40)	PE without PH before treatment (n = 39)	PE with PH before treatment (n = 34)	PE without PH after treatment (n = 39)	PE with PH after treatment (n = 34)
Global	− 23.62 ± 3.49	− 15.49 ± 3.57^△^	− 12.45 ± 4.06^△^	− 21.02 ± 5.12	− 19.53 ± 6.44*
Basal free wall	− 28.29 ± 8.43	− 17.08 ± 5.44^△^	− 16.82 ± 5.41^△^	− 26.47 ± 6.89	− 24.33 ± 7.53*
Mid free wall	− 27.49 ± 7.61	− 18.92 ± 6.03^△^	− 16.74 ± 6.28^△^	− 24.56 ± 6.37	− 22.72 ± 6.51*
Apical free wall	− 25.51 ± 9.07	− 17.11 ± 5.75^△^	− 14.88 ± 7.08^△^	− 21.48 ± 7.56	− 20.84 ± 6.42*
Basal septum	− 18.44 ± 4.26	− 12.68 ± 5.76^△^	− 9.43 ± 4.52^△^	− 16.03 ± 6.92	− 15.01 ± 5.23*
Mid septum	− 19.15 ± 4.31	− 16.31 ± 4.45*	− 11.74 ± 7.14^△^	− 17.54 ± 5.43	− 15.67 ± 6.82*
Apical septum	− 20.21 ± 5.87	− 16.44 ± 6.27*	− 13.68 ± 7.59^△^	− 18.13 ± 6.24	− 16.17 ± 7.46*

Data are expressed as $\overline{x} \pm S$

*RV* right ventricular, *PE* pulmonary embolism, *PH* pulmonary hypertension, *TTP* time to PLSS

Compared to control group, *P < 0.05, ^△^*P* < 0.01

### Correlation between strain parameters and conventional ultrasonic parameters

**Fig. 4:**
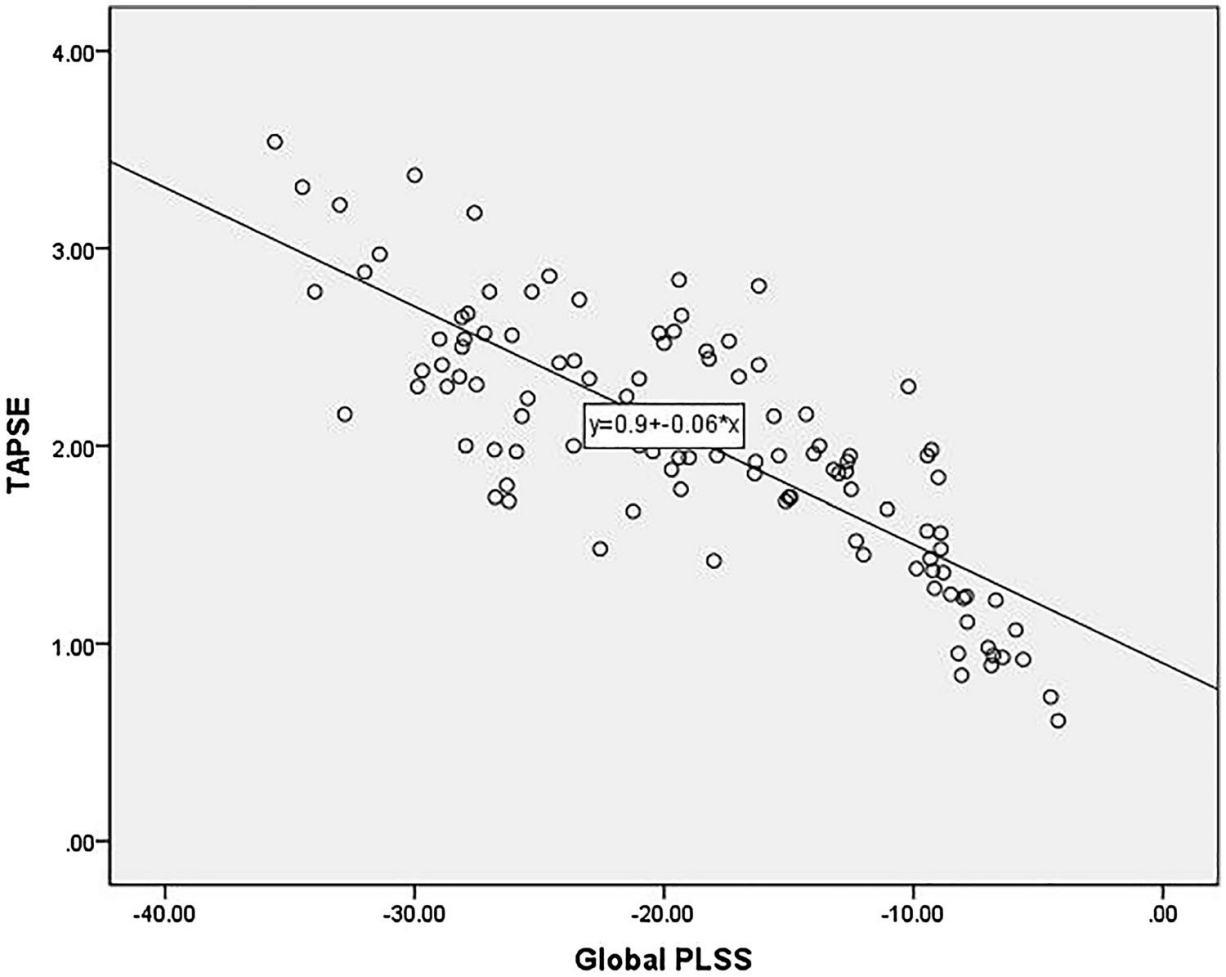
Correlative analysis showing a negative correlation between global PLSS and TAPSE

The global strain parameters PLSS of RV were positively correlated with the RVDD and RV/LV diameter ratio (correlation coefficients of 0.765 and 0.774, respectively) and negatively correlated with RVFS, RVFAC, TRPG, and TAPSE (correlation coefficients of − 0.739, − 0.752, − 0.722, and − 0.803, respectively) both before and after thrombolytic therapy (Fig. 4).

## Discussion

In this study, we used STE-derived strain parameters, including PLSS and TTP, to detect changes in global and regional RV function in PE patients with or without PH before and after thrombolytic therapy. We found that the global strain parameters for the RV were positively correlated with the RVDD and RV/LV diameter ratio and negatively correlated with the RVFS, RVFAC, TRPG, and TAPSE, demonstrating the good correlation between STE-derived parameters and conventional ultrasonic parameters for RV function. Taken together, the findings of the present study support the use of STE-derived strain parameters such as PLSS and TTP for the detection of changes in RV function during treatment for acute PE.

It has been suggested that STE may provide new quantitative indexes for regional and global cardiac function in clinical practice due to its angle-independent characteristics, which could lead to a more accurate evaluation of LV and RV function in both the long axis and short axis [6]. Strain refers to the ability of an object to deform under stress. At present, myocardial strain has been widely used in clinic as an index to evaluate myocardial mechanics. In this study, the strain parameters for the RV were obtained on the apical RV focused view, which was relatively feasible and repeatable. Previous studies have shown that STE-derived strain parameters could be successfully used to evaluate the long axis function of RV in various clinical circumstances [7]. Of note, for patients with PH, the level of pulmonary artery pressure may not fully reflect the severity of RV dysfunction as well as the efficacy of associated treatment. In this regard, RV systolic function indexes measured by ultrasound, especially the strain index reflecting the change of myocardial systolic function, may be more valuable for the evaluate of PE [8].

Our study showed that the longitudinal strain and strain rate of the RV can be used as an index for early diagnosis of RV dysfunction in patients with PE. It has been shown that the PLSS reduction is correlated with the severity of PH in these patients [9]. A typical sign of PE is the change in RV regional function, and the long axis strain of the RV free wall may reflect the RV function more accurately than the global strain of the RV [10]. Some studies showed that the movement of the middle basal segment of the RV free wall is weakened while the global systolic peak value of the RV is normal in PE patients, and these changes provided a specificity of 94% and a sensitivity of 77% for the diagnosis of RV dysfunction related to PE [11]. The mechanism of this change may include regional ischemia of the RV free wall [11]. RV free wall stain (FWS) has been proposed as a discriminator of PE patients, and adding RV FWS to the existing parameters of RV size and function could significantly improve the sensitivity and specificity for diagnosis of PE [12]. Our study also showed that the strain parameters of the RV free wall decreased more remarkably than those of the intermediate wall, which may reflect that the free wall is more sensitive to RV dysfunction related to PE. RV asynchrony in patients with PE will affect the time from the strain to peak, which could explain the findings that the global and regional TTP in patients with RV dysfunction were higher than those in the control group [13].

The increase in pulmonary vascular resistance in PE patients can cause pulmonary arterial hypertension and RV enlargement and dysfunction, which has been associated with a 2.29-fold mortality increase in PE patients [14]. The myocardium of the RV is thinner than that of the LV, which leads to poor tolerance of the RV myocardium to increased pressure overload. Additionally, the RV is more vulnerable to non-adaptive remodeling, which mainly manifests as the enlargement of the chamber and the reduction of function. Interestingly, these pathophysiological changes have been positively proportional to the degree of pulmonary artery pressure increase [15]. Subsequently, the end diastolic pressure and volume of the RV increased, whereas the LV volume decreased due to the LV septum shift left during diastole and the decrease of pulmonary reflux volume after thrombus mechanical occlusion of the pulmonary artery to a certain extent. Therefore, for PE patients, it is necessary to evaluate the RV size and RV/LV diameter ratio to comprehensively reflect the functional changes in both ventricles [16]. Generally, no significant change could be observed in the RA inner diameter when the pressure suddenly increases in acute PE, but a chronic increase in pulmonary artery pressure can lead to an increase in the RA diameter [17]. Consistently, no significant change in the RADD was observed in our patients because they all had experienced acute PE. Previous studies have shown that the RVFC and RVFAC are well correlated with the RV function assessed by myocardial magnetic resonance imaging (MRI) [18]. Our study showed that the RV global strain correlates well with the RVFC and RVFAC, indirectly confirming the accuracy of strain parameters for the assessment of RV function.

TAPSE is the displacement amplitude of the systolic wave of the lateral tricuspid annulus measured by M-mode ultrasound from the end of diastole to the end of systole, which has been related to the degree of pulmonary artery pressure overload and the degree of RV myocardial damage. TAPSE has been proposed as a prognostic factor in PE patients, conferring stronger prognostic efficacy than the RV/LV diameter ratio [19]. TAPSE reflects the movement of the long axis of the RV free wall annulus. Although TAPSE cannot fully represent the overall function of the RV, our study showed that TAPSE correlated well with global PLSS, indicating that the global and regional RV myocardial function of PE patients were both impaired. Moreover, in our study, PE patients were divided according to whether they had PH at the diagnosis of PE. Normally, PASP is less than 30 mmHg, but it may increase in PE patients to a limited degree, with the velocity of tricuspid regurgitation of 2.8 ~ 3.5 m/s [20]. The degree of the PASP increment mainly depends on the size and location of the PE, as well as whether the patient has original cardiopulmonary disease [21].

Thrombolysis is one of the main treatments for patients with PE and may effectively reduce PE and associated right heart overload, thereby attenuating the impairment of RV function. The degree of pulmonary artery pressure control plays an important role in improving the structural changes and functional lesions of the RV, because PH has been recognized as one of the most important risk factors for RV dysfunction [22]. Successful thrombolysis is generally characterized by the reduction of the RV diameter, thickening of the RV wall, and decrease in TRPG, all of which indirectly reflect the decrease in pulmonary artery pressure. In PE patients with PH, afterload of the RV was increased, and the global and regional strain may also change. Recent studies have shown that the RV global strain is well correlated with the outcome and response of treatment on PH, and the correlation with RV free wall strain is more significant than that with global strain [23]. Moreover, RV dysfunction is likely to be more severe in patients with PH, and they need more time to recover after treatment. All of these facts highlight the importance of early and effective treatments for PE patients with PH.

The study has some limitations. First, we excluded some cases with poor quality of ultrasound image and whether this had an impact on the results has not been determined. Second, we did not consider the scope of PE when grouping the patients. It is possible some patients with shock did not undergo cardiac ultrasound, and thus, the patients included in the study were likely to be hemodynamically stable. Also, due to the limited number of patients included, we were unable to divide PE patients into mild, moderate, and severe PH groups. Third, we did not use CMR to evaluate subjects, because this examination is expensive and time-consuming. Additionally, previous studies have shown that cardiac function parameters evaluated by STE are in good agreement with those evaluated by CMR. We also did not take into account the impact of gender, history of medication use, blood pressure, or diabetes on the results. However, gender distribution was matched between PE patients and controls in this study. The influence of other factors should be further evaluated in large-scale studies in the future. We did not consider whether anticoagulant therapy or other inotropic, vasopressor support had an impact on the research results, and further follow-up of patients will be included in the next research plan.

## Conclusion

STE-derived strain parameters, such as PLSS and TTP, can sensitively and accurately reflect the changes in RV global and regional stain functions in PE patients before and after thrombolysis. Moreover, these parameters showed good correlation with conventional ultrasound indexes, and thereby, can provide a supplementary ultrasonic index for clinical evaluation of curative effect. The strain parameters for the RV free wall changed more significantly when RV function was damaged. RV myocardial function in PE patients with PH is impaired more seriously. These findings support the use of STE-derived strain parameters such as PLSS and TTP for the detection of changes in RV function during the diagnosis and treatment of acute PE.

## Data Availability

The datasets generated and analyzed during the current study are not publicly available due to none of the data types requiring uploading to a public repository but are available from the corresponding author on reasonable request.

## Abbreviations

STE: Speckle tracking echocardiography
RV: Right ventricular
PE: Pulmonary embolism
PH: Pulmonary hypertension
PLSS: Peak longitudinal systolic strain
TTP: Time to PLSS
RVDD: Right ventricular diastolic diameter
RV/LV diameter ratio: Right ventricular diastolic diameter / left ventricular diastolic diameter ratio
RVFS: Right ventricular fraction shortening
RVFAC: Right ventricular fractional area change
TRPG: Tricuspid regurgitation pressure gradient
TAPSE: Tricuspid annular peak systolic excursion
CW: Continuous-wave Doppler
PASP: Pulmonary arterial systolic pressure
